# The Prognostic Value of Eosinophil Recovery in COVID-19: A Multicentre, Retrospective Cohort Study on Patients Hospitalised in Spanish Hospitals

**DOI:** 10.3390/jcm10020305

**Published:** 2021-01-15

**Authors:** María Mateos González, Elena Sierra Gonzalo, Irene Casado Lopez, Francisco Arnalich Fernández, José Luis Beato Pérez, Daniel Monge Monge, Juan Antonio Vargas Núñez, Rosa García Fenoll, Carmen Suárez Fernández, Santiago Jesús Freire Castro, Manuel Mendez Bailon, Isabel Perales Fraile, Manuel Madrazo, Paula Maria Pesqueira Fontan, Jeffrey Oskar Magallanes Gamboa, Andrés González García, Anxela Crestelo Vieitez, Eva María Fonseca Aizpuru, Asier Aranguren Arostegui, Ainara Coduras Erdozain, Carmen Martinez Cilleros, Jose Loureiro Amigo, Francisco Epelde, Carlos Lumbreras Bermejo, Juan Miguel Antón Santos, for the SEMI-COVID-19-Network

**Affiliations:** 1 Internal Medicine Department, Infanta Cristina University Hospital, 28981 Parla, Spain; irenemariacalo@gmail.com; 2 Pathology Department, Infanta Cristina University Hospital, 28981 Parla, Spain; esierrag3@yahoo.es; 3 Internal Medicine Department, La Paz University Hospital, 28046 Madrid, Spain; farnalich@salud.madrid.org; 4 Internal Medicine Department, Albacete University Hospital Complex, 02008 Albacete, Spain; jlbeato@sescam.org; 5 Internal Medicine Department, Segovia Hospital Complex, 40002 Segovia, Spain; dmonge5@hotmail.com; 6 Internal Medicine Department, Puerta de Hierro University Hospital, 28222 Majadahonda, Spain; juanantonio.vargas@uam.es; 7 Internal Medicine Department, Miguel Servet Hospital, 50009 Zaragoza, Spain; rosa.gfenoll@gmail.com; 8 Internal Medicine Department, La Princesa University Hospital, 28006 Madrid, Spain; csuarezfe@gmail.com; 9 Internal Medicine Department, A Coruña University Hospital, 15006 A Coruña, Spain; santiago.freire.castro@sergas.es; 10 Internal Medicine Department, Clinico San Carlos Hospital, 28040 Madrid, Spain; manuelmenba@hotmail.com; 11 Internal Medicine Department, Infanta Sofía Hospital, 28703 San Sebastián de los Reyes, Spain; isabel.perales@salud.madrid.org; 12 Internal Medicine Department, Dr. Peset University Hospital, 46017 Valencia, Spain; manel.madrazo@gmail.com; 13 Internal Medicine Department, Santiago Clinical Hospital, 15706 Santiago de Compostela, Spain; paulapesqueira@hotmail.com; 14 Internal Medicine Department, Nuestra Señora del Prado Hospital, 45600 Talavera de la Reina, Spain; dr990112@hotmail.com; 15 Internal Medicine Department, Ramón y Cajal University Hospital, 28034 Madrid, Spain; andres_gonzalez_garcia@hotmail.com; 16 Internal Medicine Department, Royo Villanova Hospital, 50015 Zaragoza, Spain; anxela90@gmail.com; 17 Internal Medicine Department, Cabueñes Hospital, 33394 Gijón, Spain; evamfonseca@yahoo.es; 18 Internal Medicine Department, Urduliz Alfredo Espinosa Hospital, 48610 Urdúliz, Spain; arrobahiru@gmail.com; 19 Internal Medicine Department, Santa Marina Hospital, 48004 Bilbao, Spain; ainara.coduraserdozain@osakidetza.eus; 20 Internal Medicine Department, HLA Moncloa Hospital, 28008 Madrid, Spain; cmcilleros@hotmail.com; 21 Internal Medicine Department, Moisès Broggi Hospital, 08970 Sant Joan Despí, Spain; ahores@gmail.com; 22 Internal Medicine Department, Parc Tauli Hospital, 08208 Sabadell, Spain; fepelde@gmail.com; 23 Internal Medicine Department, 12 de Octubre University Hospital, 28041 Madrid, Spain; clumbrerasb@gmail.com

**Keywords:** 2019-nCoV, SARS-CoV-2, coronavirus, COVID-19, eosinophil, prognosis

## Abstract

Objectives: A decrease in blood cell counts, especially lymphocytes and eosinophils, has been described in patients with serious Severe Acute Respiratory Syndrome Coronavirus 2 (SARS-CoV-2), but there is no knowledge of their potential role of the recovery in these patients’ prognosis. This article aims to analyse the effect of blood cell depletion and blood cell recovery on mortality due to COVID-19. Design: This work was a retrospective, multicentre cohort study of 9644 hospitalised patients with confirmed COVID-19 from the Spanish Society of Internal Medicine’s SEMI-COVID-19 Registry. Setting: This study examined patients hospitalised in 147 hospitals throughout Spain. Participants: This work analysed 9644 patients (57.12% male) out of a cohort of 12,826 patients ≥18 years of age hospitalised with COVID-19 in Spain included in the SEMI-COVID-19 Registry as of 29 May 2020. Main outcome measures: The main outcome measure of this work is the effect of blood cell depletion and blood cell recovery on mortality due to COVID-19. Univariate analysis was performed to determine possible predictors of death, and then multivariate analysis was carried out to control for potential confounders. Results: An increase in the eosinophil count on the seventh day of hospitalisation was associated with a better prognosis, including lower mortality rates (5.2% vs. 22.6% in non-recoverers, OR 0.234; 95% CI, 0.154 to 0.354) and lower complication rates, especially regarding the development of acute respiratory distress syndrome (8% vs. 20.1%, *p* = 0.000) and ICU admission (5.4% vs. 10.8%, *p* = 0.000). Lymphocyte recovery was found to have no effect on prognosis. Treatment with inhaled or systemic glucocorticoids was not found to be a confounding factor. Conclusion: Eosinophil recovery in patients with COVID-19 who required hospitalisation had an independent prognostic value for all-cause mortality and a milder course.

## 1. Introduction

In December 2019, a pneumonia of unknown origin was described in the city of Wuhan, the capital of Hubei province in China, caused by a novel coronavirus that was later named Severe Acute Respiratory Syndrome Coronavirus 2 (SARS-CoV-2) [1]. The infection was named COVID-19 (coronavirus disease 2019) in February [2], and later labelled a pandemic [3]. As a result of its global spread, overwhelming almost every healthcare system, COVID-19 has become the greatest health emergency of this century. As of 6 December 2020, nearly 66 million COVID-19 cases had been confirmed, and 1,523,656 patients had died. Initially, Europe was one of the most affected continents, with more than 19.986 million cases; Spain accounted for 1,684,647 of those cases [4].

Great effort has been made in describing the clinical and epidemiological features of COVID-19 [5,6,7], however less is known about prognostic factors [8,9,10]. Older male adults and those with diabetes, hypertension, obesity, cardiovascular disease, or chronic respiratory disease are at a greater risk of developing severe COVID-19 [6,8,9,10,11]. Some prognostic factors upon admission are lymphopenia and high levels of D-dimer (DD), lactate dehydrogenase (LDH), and C-reactive protein (CRP) [8,9,12].

Some studies have reported low total eosinophil counts in COVID-19 inpatients and persistently low eosinophil counts in more severe cases [12,13,14,15,16,17,18,19,20]; therefore, eosinopenia upon admission has been proposed as a reliable early diagnostic marker for COVID-19 infection [12,13,14]. A correlation between eosinophil recovery and radiographic and virologic recovery [15,16,17,18] as well as clinical improvement [11,12,13,15] has been more sparingly described; additionally, a worse prognosis when eosinophil levels do not recover has been suggested [11,13,15,19], whereas other studies discarded eosinopenia as a prognostic marker [12]. A meta-analysis of those reports, however, found no effect of eosinophil counts upon admission or eosinophil recovery during the course of COVID-19 [20].

The Spanish Society of Internal Medicine (SEMI, for its initials in Spanish) has launched the SEMI-COVID-19 Network, a collaborative nationwide effort to compile information on patients hospitalised with COVID-19. In a preliminary study of potential prognostic factors (not yet published), recovery from both lymphopenia and eosinopenia correlated with a lower risk of death on a multivariate analysis.

We decided to conduct a specific analysis to demonstrate whether eosinopenia or eosinophil recovery could be a prognostic factor against death due to COVID-19.

### Hypothesis and Objectives

According to our preliminary data, we hypothesised that recovery from eosinopenia could serve as an independent predictor of a favourable outcome in patients with COVID-19.

The primary aim of the study was to evaluate whether eosinophil recovery was a predictive factor of favourable progress during hospitalisation in COVID-19 patients. The secondary aims were: (a) to explore the relationship between recovery from eosinopenia and the development of acute respiratory distress syndrome (ARDS); (b) to evaluate the possible confounding effects of the use of corticosteroids in these patients; and (c) to evaluate the possible confounding effects of prior comorbidities that affect eosinophil counts.

## 2. Methods

### 2.1. Registry Design and Data Collection

The SEMI-COVID-19 Registry is an ongoing, nationwide, retrospective cohort that includes consecutive patients with a confirmed COVID-19 infection who have been hospitalised and discharged from Spanish hospitals. The registry’s characteristics have been thoroughly described in other works [21]. The full list of hospitals and collaborators is shown in Appendix A.

Inclusion criteria for the registry are age ≥18 years and first hospital discharge with a confirmed diagnosis of COVID-19. Exclusion criteria are subsequent admissions of the same patient and denial or withdrawal of informed consent. From 24 March to 29 May 2020, a total of 12,826 discharged patients were included in the registry.

Patients are treated at their attending physician’s discretion, according to local protocols and clinical judgement. Patients included in open-label clinical trials are eligible for inclusion in the registry provided that all information about treatment is available.

Data from medical records are collected retrospectively at discharge by clinical investigators all over the country, using a standardised online data capture system (DCS) described elsewhere [21]. The data collected includes many variables, collected and defined in more detail in the SEMI-COVID-19 Registry [21].

The Spanish Society of Internal Medicine is the sponsor of this registry. The researchers who coordinate the study at each hospital are SEMI members and were asked to participate in this study on a voluntary basis; they did not receive any remuneration for their participation.

The processing of personal data strictly complied with Spanish Law 14/2007, of 3 July, on Biomedical Research; Regulation (EU) 2016/679 of the European Parliament, and of the Council of 27 April 2016, on the protection of natural persons with regard to the processing of personal data and on the free movement of such data, and repealing Directive 95/46/EC (General Data Protection Regulation); and Spanish Organic Law 3/2018, of December 5, on the Protection of Personal Data and the Guarantee of Digital Rights. The SEMI-COVID-19 Registry was approved by the Provincial Research Ethics Committee of Málaga (Spain) on 27 March 2020 (Ethics Committee code: SEMI-COVID-19 27/03/20), and endorsed by the ethics committee of each participant hospital. All patients gave their informed consent. When there were biosafety concerns and/or when the patient had already been discharged, verbal informed consent was requested and noted on the medical record.

### 2.2. Study Design

A retrospective cohort study was designed in order to control for potential confounding variables. Patients included in the SEMI-COVID-19 Registry as of 31 May 2020, were selected for inclusion in this study if they had: (a) all epidemiological data recorded; (b) data on lymphocyte and eosinophil counts upon admission and on the secondary analysis at seven days after admission; and (c) onset of symptoms prior to admission. This last criterion was necessary given that the registry included nosocomial infections and, because laboratory analyses were performed upon admission and on the seventh day of hospitalisation, this ensured that the values did not correlate to clinical progress in nosocomial infections.

Descriptive analysis of the cohort and a multivariate analysis for prognostic factors was performed. Variables that have previously been demonstrated in a literature search to be correlated with eosinophil count (such as asthma or chronic corticoid use) or with COVID-19 severity or progress were considered for multivariate analysis. Variables selected for analysis included demographic variables (age, sex, race, obesity, hypertension, diabetes, alcohol abuse, tobacco use, chronic kidney disease, chronic respiratory diseases, comorbidity burden, degree of dependency, and use of inhaled or systemic corticosteroids); clinical variables (signs and symptoms upon admission, laboratory results and radiographic findings upon admission); treatment received prior to the second laboratory analysis; results of the second laboratory analysis; and clinical outcomes (specifically, pneumonia, ARDS, acute kidney injury, sepsis, ICU admission, and death).

Eosinopenia was defined as a total eosinophil count <150 × 10^6^/L upon admission. Eosinophil recovery was defined as an elevation greater than 80 × 10^6^/L on the second analysis performed on the seventh day of hospitalisation. Lymphopenia was classified into four categories: <800, 800–999, 1000–1199, and ≥1200. Lymphocyte recovery was defined as an elevation greater than 200 × 10^6^/L on the second analysis. Quick sequential organ failure assessment index (qSOFA) values were calculated from the physical findings upon admission.

All other quantitative variables were categorised as normal or abnormal (according to reference levels) upon admission. The evolution of significant values during the hospital stay were categorised as absolute elevation (for D-dimer or glycaemia) or relative elevation (for lactate dehydrogenase (LDH), aspartate aminotransferase (AST), alanine aminotransferase (ALT), and creatinine).

The STROBE Statement guidelines were followed in the conduct and reporting of the study [22].

### 2.3. Statistical Analysis

In the descriptive analysis, we summarised the epidemiological data, demographics and comorbidities, signs and symptoms upon admission, laboratory upon admission, and on the seventh day of hospitalisation, chest radiography findings, treatment received, and clinical outcomes. We performed an initial univariate analysis to determine any differences between eosinophil-recoverers and non-recoverers. We then performed a second univariate analysis to determine factors that correlated with death.

Continuous variables are expressed as means and standard deviation (SD); categorical variables are expressed as absolute values and percentages. We conducted the analysis by means of the Student’s *t*-test or ANOVA test for quantitative variables, and the Chi-squared test or Fisher’s exact test to compare differences between groups. A univariate analysis was performed to explore possible risk factors for death using binomial logistic regression.

Variables associated either with eosinophil recovery (potential confounding factors) or with death were included in a backward-stepwise multivariate logistic regression model for mortality. Survival analysis was deemed unnecessary, because there were no censored cases (each patient was discharged and the date of discharge or death was recorded in the registry), as per the design of the registry design, and time until death or discharge was not considered relevant. Quantitative variables were categorised as normal or abnormal upon admission, and significantly elevated or not significantly elevated at seven days of hospitalisation.

A secondary multivariate analysis was conducted with the composite endpoint of in-hospital death, ICU admission, or onset of moderate-to-severe ARDS.

We used SPSS (IBM Corp. Released 2017. IBM SPSS Statistics for Windows, Version 25.0. Armonk, NY: IBM Corp.) for all analyses.

## 3. Results

### 3.1. Sample Characteristics

**Figure 1:**
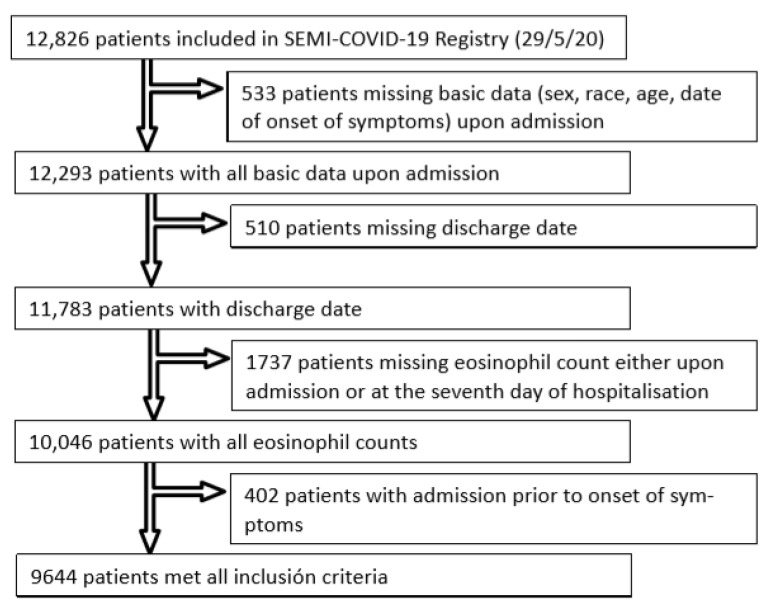
Patient Flowchart.

The SEMI-COVID-19 Registry included 12,826 patients as of 29 May 2020. Of them, 533 did not have all demographic and epidemiological data recorded (sex, age, race, and date of onset of symptoms), and thus were excluded. Another 510 patients were excluded because their discharge date was not recorded. Of the 11,783 discharged patients with all epidemiological data available, 282 were excluded because they did not have eosinophil counts upon admission, and a further 1455 were excluded for not having eosinophil counts on the seventh day of hospitalisation. Finally, 402 patients had been admitted prior to onset of symptoms and were thus also excluded. A total of 9644 patients fulfilled all inclusion criteria for this study. Of these, 3335 patients (34.6%) had eosinophil recovery, whereas 6309 patients (65.4%) did not. Figure 1 shows the flowchart for patient inclusion.

Demographic and clinical features of the study cohort are described in Table 1**.** There were differences upon admission between patients who showed eosinophil recovery and those who did not. Some important features, such as sex, obesity, or asthma, did not differ. Non-recoverers had a higher overall age and higher rates of hypertension, diabetes, chronic kidney disease (CKD), and chronic obstructive pulmonary disease (COPD). Recoverers, on the other hand, had higher comorbidity burdens and a greater degree of dependency.

Clinical presentation also differed between recoverers and non-recoverers. Recoverers had a longer duration of symptoms prior to admission, higher rates of cough and arthromyalgia, and lower rates of dyspnoea. Confusion and tachypnoea were more frequent in non-recoverers. There were no differences in temperature, heart rate, or arterial systolic tension, but oxygen saturation rates were lower in non-recoverers. A higher proportion of non-recoverers also had a qSOFA index value ≥2.

Non-recoverers had worse laboratory analysis profiles upon admission, with higher glucose, creatinine, D-dimer, and LDH levels. Lymphocyte counts were not significantly different, but eosinophil counts were lower among recoverers. Pulmonary infiltrates on radiological tests were more frequent in eosinophil recoverers.

Treatments and outcomes are summarised in Table 2. Eosinophil-recoverers were more frequently treated with hydroxychloroquine and less frequently treated with systemic or inhaled glucocorticoids. There were no differences between recoverers and non-recoverers regarding treatment with lopinavir–ritonavir, azithromycin, or low-molecular-weight heparin. All outcomes were better among eosinophil-recoverers, with lesser rates of pneumonia, ARDS, acute kidney injury, sepsis, ICU admission, and death. Notably, 94.8% of eosinophil-recoverers were discharged alive (vs 77.4% in non-recoverers, *p* < 0.001), 91.3% were discharged without requiring ICU admission (vs 71.1% in non-recoverers, *p* < 0.001), and 85.8% were discharged with neither ICU admission nor onset of ARDS during hospitalisation (vs 65.5% in non-recoverers, *p* < 0.001).

### 3.2. Outcomes

Variables that correlated with either mortality or eosinophil recovery upon univariate analysis (Table 3), as well as potential confounding factors, were introduced into a multivariate analysis using mortality as the dependent variable. Several cut-off points for categorisation were checked for sensitivity analysis. The final regression model is summarised in Table 4, and shows that eosinophil recovery was independently associated with lower mortality, with an OR of 0.234 (95% CI, 0.154 to 0.354). Initial eosinopenia was not found to be significant in the analysis. A lymphocyte count lower than 800 × 10^6^/L upon admission was predictive of death, but neither further categorisation of lymphocyte value ranges nor lymphocyte recovery were. Corticosteroid treatment was not found to correlate with death in our analysis, whereas both hydroxychloroquine and azithromycin correlated with a lower mortality rate. Notably, both elevated ALT upon admission and at seven days of hospitalisation correlated with a lower mortality rate. More studies are needed to clarify this finding.

A secondary multivariate analysis was performed for the secondary composite endpoint of in-hospital death, ICU admission, or onset of ARDS during hospitalisation (Table 5). After controlling for other variables, eosinophil recovery was found to correlate with a lower chance of worse progress (OR 0.474; 95% CI, 0.383–0.586).

## 4. Discussion

Our study shows that eosinophil recovery has a positive prognostic impact in COVID-19 that is independent of previous lymphocyte or eosinophil levels and previous use of systemic or inhaled corticosteroids. To the best of our knowledge, this work is the first instance where this prognostic factor has been thoroughly described in a large cohort.

Abnormal laboratory values in patients with COVID-19, in particular low levels of lymphocytes, have been described in several studies, but less emphasis has been placed on low levels of eosinophils [8,9,12,16,19]. Lymphocyte depletion has been shown to have diagnostic value, along with prognostic value shown in various studies, albeit inconsistently. The recovery of lymphocytes and eosinophils has been studied to a lesser degree than the implications of their initial values [19]. The first descriptions of eosinophil depletion came from small series [16,18], and eosinopenia upon admission was proposed as a reliable early diagnostic marker for SARS-COV-2 infection [12,13,14]. Eosinopenia has already been described by Echevarria et al. [23] as an independent predictor of death in non-COPD patients with pneumonia, regardless of corticosteroid use. In COVID-19 pneumonia, eosinophil recovery has also been proposed to be a marker for clinical improvement, and sustained eosinopenia a marker of poorer prognosis [11,13,15]. Du Yu also found a correlation with higher viral loads [11]. However, in a series of 414 patients, eosinopenia was not related to prognosis [12], and a previous meta-analysis of 294 subjects [20] had showed that eosinophil levels made no difference in the progress and mortality of patients with COVID-19.

The recovery of lymphocytes and eosinophils has been studied to a lesser degree than the implications of their initial values [14].

In our cohort, which comprised 9644 patients, a profound degree of eosinopenia was found upon diagnosis of COVID-19, with a higher mortality rate observed in patients with eosinopenia patients than patients without eosinopenia (16.7% vs. 13.2%, *p* = 0.04). Furthermore, eosinophil recovery was associated with higher survival rates, as was found by Sun et al. [19]. However, these findings could have been due to a number of confounding factors, the most obvious being that comorbidities or immunosuppressive drugs (used predominantly in more severe cases) could have been responsible for the prolonged eosinopenia, and thus eosinophil recovery would be a marker of other previous prognostic factors. Another explanation could be that eosinophil levels and eosinophil recovery are parallel to lymphocyte levels, representing the same degree of immune response to SARS-CoV-2. The most obvious potential confounding factor is prior use of glucocorticoids, which have been widely described as a cause of eosinopenia by means of medullary retention [12]. For this reason, we designed our study to control for the use of systemic or inhaled glucocorticoids both before and during hospitalisation as a potential confounding factor in sustained eosinopenia and COVID-19 progress.

The multivariate analysis showed no effects of chronic or acute use of corticosteroids, asthma, or other diseases that affect eosinophil levels on the predictive capacity of plasmatic eosinophils. In our analysis, asthma or pulmonary infiltrates on radiological tests did not significantly correlate with mortality and were eliminated from the model. The elevation of eosinophils was found to be associated with a better prognosis and lower mortality rate, with an OR of 0.234 (95% CI, 0.154 to 0.354), independently of previous use of glucocorticoids. Our results are in contrast to the conclusions of the meta-analysis by Lippi et al. [20] or the series of Mamta Soni [12] and corroborate the results of other works [11,13,14,15] where the recovery of eosinopenia is proposed as a potential prognostic factor in COVID-19. All these findings emphasise the incompletely explored role of eosinophils, either as an immunological side effect of the SARS-COV2 virus or as an immunomodulatory factor in COVID-19. Our results could be explained by either distinct initial inflammatory responses to SARS-CoV-2, with an initial predisposition towards a Th2 response, or by different inflammatory evolutions, with an immune recovery with modification from an initial Th1 inflammatory response to a Th2 response [24], or indeed both of them simultaneously.

It has been proposed that SARS-COV2 infections induces eosinopenia. Proposed mechanisms for SARS-COV-2-induced eosinopenia could involve a diminished release of eosinophils from the bone marrow, a blockade in eosinophilopoiesis, direct eosinophil apoptosis induced by dysfunctional type I IFNs response during virus infection, or all of them combined [17]. Eosinophil recovery could thus simply be a marker of lesser virological activity, but this is not probable because it has been found to precede the negativization of nucleic acid assays by five days [15].

Eosinophil recovery could be a marker of a different inflammatory pathways associated with mortality. Several studies have demonstrated the key role of eosinophils in the initiation and maintenance of inflammation through stimulation of a Th2 inflammatory response, as well as their direct association with inflammatory diseases such as asthma [25,26,27,28,29]. Curiously, asthma, which was initially suspected to be a risk factor in COVID-19, has been consistently shown to have a protective role in various cohorts [4,9,17], except for severe asthma, which may be neutrophilic asthma not mediated by a Th2 response. If an underlying Th2 response is involved in eosinophil recovery, it would be expected that we would find a higher proportion of asthmatic patients amongst eosinophil-recoverers and higher levels of eosinophils upon admission. However, in our series, eosinopenia was more severe in eosinophil-recoverers and thus does not suggest a Th2 response prior to admission.

On the other hand, patients with obesity and type 2 diabetes mellitus are known to have a higher Th1 inflammatory response [30,31] and to have a worse COVID-19 prognosis [6,8,9,10,11,32]. Both greater eosinopenia and lower recovery of eosinophil counts could simply be markers of these previous comorbidities; both diabetes and obesity were more prevalent among non-recoverers. Our univariate analysis confirmed higher mortality rates amongst patients with obesity and diabetes, but this effect disappeared in the multivariate analysis. Therefore, it could well be the other way round: instead of eosinophil recovery being a surrogate for lower diabetes rates, the latter could be a deleterious factor because it implies an intrinsic Th1 response, leading to a worse prognosis for COVID-19.

Another possible immunological explanation for the role of eosinophils could be that, regardless of the initial response to SARS-CoV-2 infection, eosinophil recovery represents a marker of immune recovery. This could also be due to a non-specific pathway or to Th2 switching. Were it due to non-specific recovery, it would merely be a marker of good progress with no special immunological significance and should be paralleled or followed by lymphocyte recovery. Our study shows that lymphocyte recovery at the seventh day of hospitalisation is not an independent marker of a good prognosis, whereas Sun et al. [19] found an elevation in lymphocyte counts in less severe cases, albeit starting later than eosinophil recovery. Our database only includes two laboratory analyses (upon admission and on the seventh day); therefore, it was not possible for us to ascertain whether a later lymphocyte recovery exists or if it has prognostic implications. Regardless, a marker of a good prognosis after the seventh day of hospitalisation is probably less useful than an earlier predictor would be.

On the other hand, eosinophil recovery could be a marker of Th2 switching [33,34,35], thus possibly indicating a different inflammatory response to SARS-CoV-2 and leading to less susceptibility to ARDS. This is a highly interesting explanation that should be studied further, because it could well lead to new therapeutic strategies for COVID-19.

Different immunological profiles [33,34,35] have been described in other inflammatory diseases of both autoimmune and infectious origin. The ones most commonly described are the Th1 pathways (involving the so-called Th1 cytokines of IL-12, IFN, and TNF-α, leading to activation of CD8+ T cells and classically activated macrophages), the Th2 pathways (mediated by IL-4, IL-5, and IL-13, leading to activation of eosinophils, alternatively activated macrophages, and B-lymphocytes), and the Th17 pathways (mediated by IL-1, IL-6, and the inflammasome, leading to IL-17 and IL-22) [34,35,36]. In COVID-19, cytokine elevation has been described as a marker of worse progress (higher ARDS and death rates), with involvement serum levels of both IL-1 and IL-6. These patients probably develop a Th1–Th17 response to the infection. A depletion of Treg lymphocytes, which are crucial for the negative regulation of proliferation and inflammation, has been described in COVID-19 patients, especially in more severe cases [37]. There is no knowledge of the mechanism of lung inflammation, because live biopsies have not been described to date. Autopsies after ICU death have shown low-grade inflammation and high rates of local microthrombosis [38], but this may be the advanced, terminal stage of a previous inflammatory injury. Different inflammatory pathways could explain the different progress observed amongst COVID-19 patients. It may not be a question of whether an inflammatory response is provoked, but rather which inflammatory response is provoked. Lessening cytokine dysregulation with immunosuppressants has already been attempted. Perhaps efforts towards inducing a Th2 response could improve patient prognosis but, to our knowledge, there is no pharmacological pathway to do so.

In our study, we explored other changes in the laboratory findings over the course of a patient’s disease. Our multivariate model showed the significance of ALT, LDH, creatinine, and D-dimer elevation. Another finding in our study was the protective effect of both hydroxychloroquine and azithromycin observed in our sample. These findings should be interpreted cautiously so as not to fall in “*Table 2 Fallacy*” [39]; our study was not designed to control for confounding factors of renal or hepatic function.

Finally, we also explored the composite endpoint of in-hospital death, ICU admission, or onset of ARDS during patients’ hospital stays. Eosinophil recovery also correlated favourably with this outcome, with an OR of 0.474 (95% CI, 0.383 to 0.586), meaning that not only was death less frequent among eosinophil-recoverers, but a milder course could be predicted. This is highly important, because if eosinophil recovery is confirmed as a marker of a good prognosis, it could be used to guide decisions regarding discharge in otherwise stable patients. In the context of a pandemic, this could help alleviate the strain on healthcare systems by identifying potential candidates for early discharge.

Among the strengths of the SEMI-COVID-19 Registry and its consequent studies are its multicentre, nationwide design, along with the large number of patients included, which provides strong statistical power for confirming hypotheses. However, for the same reason, all the studies based on the SEMI-COVID-19 Registry have common limitations. Only inpatients were included; therefore, it is not possible to extrapolate our results to outpatients. Information bias could be introduced by either the large number of researchers involved or variability in the availability of data from each hospital. Finally, selection bias could be introduced given the voluntary participation of each centre.

Our study was designed to control for possible confounding factors for abnormal eosinophil values, but some of them could not be controlled due to the nature of the data available in the registry. Transfusion of blood products was not recorded and thus this information was not available for study. The influence of the stress response and hormonal treatment were also not recorded, but should be taken into account when assessing haematological parameters. Bacterial coinfection during, or superinfection after, contracting SARS-CoV-2 could have led to different immune responses. Neither thorough cytokine profiles nor lymphocyte subset panels were obtained, because this registry reflects usual clinical practice and not basic research, therefore inflammatory pathways were not studied. Further research is needed to overcome these limitations.

In conclusion, eosinophil recovery at the seventh day of hospitalisation was a predictor of a good prognosis in COVID-19 inpatients from our cohort, and warrants further research.

## 5. Conclusions

Eosinophil recovery, independently of treatments administered and the patients’ underlying condition, was a marker of good prognosis in our cohort. If confirmed, it could help in making decisions about safe discharge.

More studies are needed to assess whether eosinophil recovery is a marker of general immune recovery or of a different immunological response profile to the infection.

## Figures and Tables

**Table 1:** Baseline demographic and clinical features upon admission of patients with eosinophil recovery during the course of COVID-19 (Recoverers) and those who did not (Non-Recoverers).

KERRYPNX	Recoverers	Non-Recoverers	*p*
Demographics			
Patients	3335 (34.6)	6309 (65.4)	
Age (years) (*n* = 9644)	63.85 ± 15.57	67.52 ± 16.0	<0.001
Gender (male) (*n* = 5509)	1904 (57.1)	3605 (57.2)	0.479
Race/ethnicity (*n* = 9489)			<0.001
Caucasian	2797 (85.3)	5603 (90.2)	
Latino/a	406 (12.4)	505 (8.1)	
African/Black	15 (0.5)	26 (0.4)	
Alcohol abuse (*n* = 9394)	126 (3.9)	298 (4.8)	0.036
Tobacco use (*n* = 9218)			
Current smoker	156 (4.9)	332 (5.5)	
Former smoker	741 (23.3)	1562 (25.8)	0.009
Degree of dependency (*n* = 9527)			<0.001
Independent or mild	138 (4.2)	439 (7.0)	
Moderate	175 (5.3)	587 (9.4)	
Severe	2986 (90.5)	5202 (83.5)	
Cardiovascular risk factors			
Hypertension (*n* = 9628)	1510 (45.3)	3222 (51.2)	<0.001
Diabetes mellitus (*n* = 9612)	558 (16.8)	1259 (20.0)	<0.001
Obesity (*n* = 8785)	670 (22.0)	1240 (21.6)	0.346
Respiratory diseases			
COPD (*n* = 9619)	159 (4.8)	476 (7.6)	<0.001
Asthma (*n* = 9617)	245 (7.4)	504 (8.0)	0.263
Chronic kidney failure *(n* = 9616)	132 (4.0)	410 (6.5)	<0.001
Comorbidity (*n* = 9366)			<0.001
No comorbidities	373 (11.5)	1158 (18.9)	
Mild	329 (10.2)	850 (13.9)	
Severe	2538 (78.3)	4118 (67.2)	
Previous chronic drug therapy			
Chronic treatment with systemic corticoids (*n* = 9618)	82 (2.5)	313 (5.0)	<0.001
Chronic treatment with inhaled corticoids (*n* = 9578)	262 (7.9)	657 (10.5)	<0.001
Symptoms			
Time from onset of symptoms (days) (*n =* 9644)	7.4 (7.7)	6.7 (4.8)	<0.001
Cough (*n* = 9619)			0.011
No	749 (22.5)	1003 (25.2)	
Dry	2044 (61.4)	3703 (58.9)	
Productive	534 (16.1)	1586 (15.9)	
Dyspnoea (*n* = 9599)	1850 (55.8)	3683 (58.6)	0.007
Arthromyalgia (*n* = 9539)	1172 (35.5)	1910 (30.6)	<0.001
Asthenia (*n* = 9513)	1501 (45.6)	2705 (43.5)	0.048
Anorexia (*n* = 9482)	626 (19.1)	1265 (20.4)	0.123
Fever at home (*n* = 9608)			0.065
<37 °C	448 (13.5)	915 (14.6)	
37.0–37.9 °C	651 (19.6)	1307 (20.8)	
>38.0 °C	2230 (67.0)	4057 (64.6)	
Physical examination at admission			
Confusion (*n* = 9530)	223 (6.8)	740 (11.9)	<0.001
Tachypnoea (>20 brpm) (*n* = 9394)	848 (26.0)	2029 (33.1)	<0.001
SBP (mmHg) (*n* = 9252)	128.5 ± 20.28	128.8 ± 21.3	0.538
Heart rate (bpm) (*n* = 9351)	88.8 ± 16.90	88.6 ± 17.5	0.581
Temperature (°C) (*n* = 9345)	37.1 ± 0.98	37.1 ± 0.98	0.253
Oxygen saturation (*n* = 9410)	93.8 ± 4.45	92.9 ± 5.7	<0.001
Saturation <95%	1550 (47.5)	3197 (52)	<0.001
Lung auscultation			
Crackles (*n* = 9416)	1726 (53.1)	3290 (53.3)	0.854
Wheezing (*n* = 9414)	143 (4.4)	425 (6.9)	<0.001
qSOFA score (*n* = 9644)			<0.001
0–1	3196 (95.8)	5799 (91.9)	
2–3	139 (4.2)	510 (8.1)	
Additional tests			
Radiological findings			
Interstitial pulmonary infiltrates (*n* = 9600)			<0.001
No pulmonary infiltrates	348 (10.5)	853 (13.6)	
Unilateral pulmonary infiltrates	726 (21.9)	1308 (20.8)	
Bilateral pulmonary infiltrates	2247 (67.7)	4118 (65.5)	
Laboratory findings upon admission			
PO2/FiO2 ratio (mmHg) (*n* = 4859)	303.3 ± 94.7	288.5 ± 98.6	<0.001
Leukocytes × 10^6^/L (*n* = 9644)	7262 ± 5002	7192 ± 5562	0.538
Eosinophils × 10^6^/L (*n* = 9644)	18.25 ± 64.13	37.45 ± 107.64	<0.001
Eosinopenia <150 × 10^6^/L	3252 (97.5)	5906 (93.6)	<0.001
Lymphocytes × 10^6^/L *(n* = 9644)	1126 ± 1562	1098 ± 1805	0.443
Lymphopenia <800 × 10^6^/L	939 (28.2)	2312 (36.6)	<0.001
Lymphopenia <800 × 10^6^/L			
Lymphopenia <800 × 10^6^/L			
Neutrophils × 10^6^/L (*n* = 9644)	5230 ± 2927	5192 ± 3382	0.583
CRP (mg/L) (*n* = 9285)	82.2 ± 80	85.2 ± 86.3	0.097
Glucose (mg/dL) (*n* = 9368)	123.7 ± 52.9	127.3 ± 57.7	0.003
Creatinine (mg/dL) (*n* = 9614)	1.0 ± 0.69	1.11 ± 0.86	<0.001
Urea (mg/dL) (*n* = 7713)	41.8 ± 31.5	48.3 ± 36.6	<0.001
LDH (U/L) (*n* = 8448)	341.2 ± 155.1	355.7 ± 179.3	<0.001
AST (U/L) (*n* = 7616)	47.4 ± 48.3	47.7 ± 59.1	0.847
ALT (U/L) (*n* = 9120)	42.2 ± 42.0	41.2 ± 52.4	0.373
D-dimer (ng/mL) (*n* = 7567)	1354.8 ± 5157	1619.7 ± 5548	0.043

COPD: chronic obstructive pulmonary disease. Comorbidity was measured using the Charlson Comorbidity Index. brpm: breaths per minute. SBP: systolic blood pressure. mmHg: millimetres of mercury. bpm: beats per minute. qSOFA: quick sequential organ failure assessment. CRP: C-reactive protein. LDH: lactate dehydrogenase. AST: aspartate aminotransferase. ALT: alanine aminotransferase. Categorical variables are expressed as N (%), quantitative variables as mean ± SD.

**Table 2:** Management and progress during hospitalisation of patients with and without eosinophil elevation.

	Recoverers	Non-Recoverers	*p*
Treatment Received			
LPV/r (*n* = 9606)	2151 (64.6)	4047 (64.5)	0.868
Hydroxychloroquine (*n* = 9617)	3016 (90.5)	5432 (86.4)	<0.001
Systemic corticosteroids (*n* = 9644)	458 (13.7)	2003 (31.7)	<0.001
Tocilizumab (*n* = 9578)	274 (8.2)	664 (10.6)	<0.001
Azithromycin (*n* = 9592)	2079 (62.6)	3911 (62.4)	0.501
Inhaled corticosteroids (*n* = 9497)	164 (5.0)	400 (6.5)	0.004
LMWH (*n* = 9564)	2804 (84.5)	5256 (84.1)	0.611
Outcomes			
Pneumonia (*n* = 9605)	257 (7.7)	791 (12.6)	<0.001
ARDS (*n* = 9595)			<0.001
No	2605 (78.3)	3933 (62.7)	
Mild	299 (9.0)	547 (8.7)	
Moderate	156 (4.7)	531 (8.5)	
Severe	266 (8.0)	1258 (20.1)	
Acute kidney failure (*n* = 9613)	315 (9.5)	985 (15.7)	<0.001
Sepsis (*n* = 9604)	102 (3.1)	462 (7.4)	<0.001
ICU admission (*n* = 9636)	179 (5.4)	678 (10.8)	<0.001
Length of hospital stay (days) (*n* = 9644)	11.0 ± 7.8 (*)	11.5 ± 9.2 (*)	<0.001
Death (in-hospital) (*n* = 9644)	172 (5.2)	1423 (22.6)	<0.001
Composite endpoint (in-hospital death or ICU admission or ARDS) (*n* = 9612)	472 (14.2)	2170 (34.5)	<0.001
Discharge	3163 (94.8)	4886 (77.4)	<0.001
without ICU admission	3040 (91.3)	4484 (71.1)	
without ICU admission or ARDS	2852 (85.8)	4118 (65.5)	

LPV/r: lopinavir/ritonavir. LMWH: low-molecular-weight heparin. ARDS: acute respiratory distress syndrome. ICU: intensive care unit. Categorical variables are expressed as N (%), quantitative variables (*) as mean ± SD.

**Table 3:** Univariate analysis of mortality. Quantitative variables are expressed as mean ± SD in survivors and non-survivors. Categorical variables are expressed as mortality in N (%) for factor present and factor absent. For categorical variables with more than two categories, mortality is provided for each category as N (%).

	Non-Survivors	Survivors	*p*
Age (years)	78.7 ± 10.5	63.8 ± 15.7	<0.001
Time from onset of symptoms at admission (days)	5.7 ± 5.0	7.2 ± 6.1	<0.001
Length of hospital stay (days)	10.5 ± 9.4	11.5 ± 8.6	<0.001
Factor	Mortality when present	Mortality when absent	
Demographics			
Male gender	1011 (18.4)	581 (14.1)	<0.001
Caucasian race/ethnicity	1503 (17.9)	72 (6.6)	<0.001
Alcohol abuse	86 (20.3)	1468 (16.4)	0.034
Tobacco use	585 (21.0)	936 (14.6)	<0.001
Moderate or severe dependency	531 (39.7)	1043 (12.7)	<0.001
Hypertension	1115 (13.6)	477 (9.7)	<0.001
Obesity	359 (18.8)	1067 (15.5)	0.001
Diabetes mellitus	480 (26.4)	1111 (14.3)	<0.001
COPD	200 (31.5)	1390 (15.5)	<0.001
Asthma	89 (11.9)	1500 (16.9)	<0.001
Chronic kidney disease	200 (36.9)	1389 (15.3)	<0.001
Moderate or severe comorbidity	523 (34.2)	1023 (13.1)	<0.001
Chronic treatment with systemic corticosteroids	113 (28.6)	1476 (16.0)	<0.001
Chronic treatment with inhaled corticosteroids	208 (22.6)	1372 (15.8)	<0.001
Symptoms			
Cough	1093 (15.0)	494 (21.2)	<0.001
Dyspnoea	1100 (19.9)	485 (11.9)	<0.001
Arthromyalgia	309 (10.0)	1261 (19.5)	<0.001
Asthenia	640 (15.2)	924 (17.4)	0.004
Anorexia	373 (19.7)	1182 (15.6)	<0.001
Fever at home	1266 (15.4)	315 (23.1)	<0.001
Physical examination			
Confusion	439 (45.6)	1135 (13.2)	<0.001
Tachypnoea >20 brpm	848 (29.5)	699 (10.7)	<0.001
Hypotension (<90 mmHg)	56 (36.4)	1492 (16.4)	<0.001
Tachycardia >100 bpm	335 (16.0)	1215 (16.8)	0.383
Temperature >37.7 °C	431 (17.5)	1106 (16.1)	0.115
Oxygen saturation via pulse oximetry (%)			<0.001
Normal (>94%)	375 (8.0)		
Hypoxemia (90–94%)	551 (16.9)		
Desaturation (<90%)	628 (42.2)		
Crackles	943 (18.8)	603 (13.7)	<0.001
Wheezing	152 (26.8)	1392 (15.7)	<0.001
qSOFA score ≥2	333 (51.3)	1262 (14.0)	<0.001
Findings upon admission			
Pulmonary infiltrates on radiological tests	1417 (16.9)	173 (14.4)	0.320
Eosinophils (×10^6^/L)			0.084
>300	26 (15.1)		
150–299	38 (12.1)		
<150	1531 (16.7)		
Eosinopenia <150 × 10^6^/L	1531 (16.7)	64 (13.2)	0.040
Lymphocytes (×10^6^/L)			<0.001
>1200	337 (10.9)		
1000–1199	184 (12.1)		
800–999	269 (15.0)		
<800	805 (24.8)		
Lymphopenia <800 × 10^6^/L	805 (24.8)	790 (12.4)	<0.001
Basal glucose >125 mg/dL	792 (27.4)	761 (11.8)	<0.001
High creatinine (>1.4 mg/dL)	568 (42.2)	1025 (12.4)	<0.001
LDH >360 U/L	702 (23.1)	585 (10.8)	<0.001
AST >60 U/L	322 (21.4)	893 (14.6)	<0.001
ALT >60 U/L	193 (13.0)	1246 (16.3)	0.001
D-dimer (ng/mL)			<0.001
<500	269 (8.9)		
500–999	289 (12.4)		
>1000	535 (24.1)		
D-dimer >1000 ng/mL	535 (24.1)	558 (10.4)	<0.001
Treatment			
Lopinavir/ritonavir	929 (15.0)	656 (19.2)	<0.001
Hydroxychloroquine	1246 (14.7)	341 (29.2)	<0.001
Systemic corticosteroids	532 (21.6)	1063 (14.8)	<0.001
Tocilizumab	214 (22.8)	1372 (15.9)	<0.001
Azithromycin	914 (15.3)	664 (18.4)	<0.001
Inhaled corticosteroids	117 (20.7)	1446 (16.2)	0.005
Low-molecular-weight heparin	1324 (16.4)	252 (16.8)	0.753
Findings during progress			
Eosinophils increased >80 × 10^6^/L	172 (5.2)	1423 (22.6)	<0.001
Lymphocyte increased >200 × 10^6^/L	288 (6.2)	1307 (26.1)	<0.001
LDH increased >50%	349 (48.9)	760 (11.0)	<0.001
Creatinine increased >50%	224 (62.0)	1350 (14.7)	<0.001
D-dimer increased >500 ng/mL	339 (26.8)	491 (9.8)	<0.001
Glycaemia increased >100 mg/dL	136 (42.6)	1353 (15.6)	<0.001
AST increased 3×	72 (24.4)	1045 (15.5)	<0.001
ALT increased 3×	125 (13.4)	1228 (15.9)	0.040

COPD: chronic obstructive pulmonary disease. Comorbidity was measured using the Charlson Comorbidity Index. brpm: breaths per minute. mmHg: millimetres of mercury. bpm: beats per minute. qSOFA: quick sequential organ failure assessment. CRP: C-reactive protein. LDH: lactate dehydrogenase. AST: aspartate aminotransferase. ALT: alanine aminotransferase. Quantitative variables are expressed as mean ± SD in survivors and non-survivors. Categorical variables are expressed as mortality in N (%) for factor present and factor absent. For categorical variables with more than two categories, mortality is provided for each category as N (%).

**Table 4:** Multivariate analysis of mortality. The effect of each factor is expressed as an adjusted odds ratio (CI 95%).

	Adjusted OR	*p*
Demographics		
Age (years)	1.050 (1.036 to 1.065)	0.000
Gender (female)	0.644 (0.471 to 0.881)	0.006
Hypertension	1.320 (0.996 to 1.816)	0.087
Moderate-to-severe dependency	2.250 (1.515 to 3.342)	0.000
Clinical manifestations at admission		
Cough	0.670 (0.483 to 0.929)	0.016
Confusion	1.718 (1.149 to 2.569)	0.008
Tachypnoea	1.894 (1.397 to 2.566)	0.000
Wheezing	1.597 (0.966 to 2.639)	0.068
Desaturation		
Saturation 90–94%	1.701 (1.196 to 2.420)	0.003
Saturation <90%	4.594 (3.084 to 6.843)	0.000
Treatment during hospitalisation		
Hydroxychloroquine	0.662 (0.432 to 1.013)	0.057
Azithromycin	0.647 (0.475 to 0.881)	0.006
Laboratory findings at admission		
Creatinine >1.4 at admission	1.564 (1.103 to 2.219)	0.012
LDH >360 at admission	2.450 (1.757 to 3.416)	0.000
AST >60 at admission	2.462 (1.637 to 3.704)	0.000
ALT >60 at admission	0.444 (0.274 to 0.720)	0.001
Glycaemia >125 at admission	1.405 (1.045 to 1.889)	0.024
Lymphopenia <800 × 10^6^/L at admission	1.452 (1.086 to 1.942)	0.012
Laboratory findings on the seventh day of hospitalisation		
Eosinophil counts increased >80 × 10^6^/L	0.234 (0.154 to 0.354)	0.000
LDH increased >1.5×	10.614 (7.101 to 15.867)	0.000
Creatinine increased >1.5×	6.032 (3.528 to 10.315)	0.000
D-dimer increased >500	2.341 (1.718 to 3.189)	0.000
ALT increased >3×	0.536 (0.321 to 0.894)	0.017

**Table 5:** Multivariate analysis of the composite endpoint of in-hospital death or ICU admission or moderate-to-severe ARDS. The effect of each factor is expressed as an adjusted odds ratio (CI 95%).

	Adjusted OR	*p*
Demographics		
Race (Caucasian)	0.715 (0.528 to 0.969)	0.030
Clinical manifestations at admission		
Duration of symptoms at admission (days)	0.962 (0.942 to 0.983)	0.000
Cough	1.070 (0.856 to 1.337)	0.055
Confusion	1.783 (1.320 to 2.409)	0.000
Tachypnoea >20 brpm	2.057 (1.697 to 2.495)	0.000
Wheezing	1.402 (0.987 to 1.991)	0.059
Fever	1.375 (1.125 to 1.681)	0.002
Desaturation		
Saturation 90–94%	1.694 (1.377 to 2.084)	0.000
Saturation <90%	4.856 (3.730 to 6.322)	0.000
Treatment during hospitalization		
Hydroxychloroquine	0.684 (0.504 to 0.928)	0.015
Corticosteroids	1.634 (1.348 to 1.979)	0.000
Laboratory findings at admission		
Creatinine >1.4 at admission	1.497 (1.162 to 1.928)	0.002
D-dimer >1000 at admission	1.226 (1.006 to 1.495)	0.044
LDH >360 at admission	2.306 (1.907 to 2.790)	0.000
Glycaemia >125 at admission	1.386 (1.143 to 1.681)	0.001
Lymphopenia <800 × 10^6^/L at admission	1.541 (1.222 to 1.944)	0.000
Any pulmonary infiltrates	2.306 (1.601 to 3.321)	0.000
Laboratory findings on the seventh day of hospitalisation		
Eosinophil counts increased >80 × 10^6^/L	0.474 (0.383 to 0.586)	0.000
LDH increased >1.5×	6.437 (4.779 to 8.669)	0.000
Creatinine increased >1.5×	3.485 (2.160 to 5.620)	0.000
D-dimer increased >500	2.643 (2.155 to 3.241)	0.000
Glycaemia increased >100 mg/dL	1.661 (1.083 to 2.548)	0.020

## Data Availability

Data is contained within the article.

## Appendix A

List of the SEMI-COVID-19 Network members

Coordinator of the SEMI-COVID-19 Registry: José Manuel Casas Rojo.

SEMI-COVID-19 Scientific Committee Members: José Manuel Casas Rojo, José Manuel Ramos Rincón, Carlos Lumbreras Bermejo, Jesús Millán Núñez-Cortés, Juan Miguel Antón Santos, Ricardo Gómez Huelgas.

SEMI-COVID-19 Registry Coordinating Center: S & H Medical Science Service.

Members of the SEMI-COVID-19 Group

H. U. 12 de Octubre. Madrid

Paloma Agudo de Blas, Coral Arévalo Cañas, Blanca Ayuso, José Bascuñana Morejón, Samara Campos Escudero, María Carnevali Frías, Santiago Cossio Tejido, Borja de Miguel Campo, Carmen Díaz Pedroche, Raquel Diaz Simon, Ana García Reyne, Lucia Jorge Huerta, Antonio Lalueza Blanco, Jaime Laureiro Gonzalo, Carlos Lumbreras Bermejo, Guillermo Maestro de la Calle, Barbara Otero Perpiña, Diana Paredes Ruiz, Marcos Sánchez Fernández, Javier Tejada Montes.

H. U. Gregorio Marañón. Madrid

Laura Abarca Casas, Álvaro Alejandre de Oña, Rubén Alonso Beato, Leyre Alonso Gonzalo, Jaime Alonso Muñoz, Crhistian Mario Amodeo Oblitas, Cristina Ausín García, Marta Bacete Cebrián, Jesús Baltasar Corral, Maria Barrientos Guerrero, Alejandro Bendala Estrada, María Calderón Moreno, Paula Carrascosa Fernández, Raquel Carrillo, Sabela Castañeda Pérez, Eva Cervilla Muñoz, Agustín Diego Chacón Moreno, Maria Carmen Cuenca Carvajal, Sergio de Santos, Andrés Enríquez Gómez, Eduardo Fernández Carracedo, María Mercedes Ferreiro-Mazón Jenaro, Francisco Galeano Valle, Alejandra Garcia, Irene Garcia Fernandez-Bravo, María Eugenia García Leoni, Maria Gomez Antunez, Candela González San Narciso, Anthony Alexander Gurjian, Lorena Jiménez Ibáñez, Cristina Lavilla Olleros, Cristina Llamazares Mendo, Sara Luis García, Víctor Mato Jimeno, Clara Millán Nohales, Jesús Millán Núñez-Cortés, Sergio Moragón Ledesma, Antonio Muiño Miguez, Cecilia Muñoz Delgado, Lucía Ordieres Ortega, Susana Pardo Sánchez, Alejandro Parra Virto, María Teresa Pérez Sanz, Blanca Pinilla Llorente, Sandra Piqueras Ruiz, Guillermo Soria Fernández-Llamazares, María Toledano Macías, Neera Toledo Samaniego, Ana Torres do Rego, Maria Victoria Villalba Garcia, Gracia Villarreal, María Zurita Etayo.

Hospital Universitari de Bellvitge. L’Hospitalet de Llobregat

Xavier Corbella, Narcís Homs, Abelardo Montero, Jose María Mora-Luján, Manuel Rubio Rivas.

H. U. La Paz-Cantoblanco-Carlos III. Madrid

Jorge Álvarez Troncoso, Francisco Arnalich Fernández, Francisco Blanco Quintana, Carmen Busca Arenzana, Sergio Carrasco Molina, Aranzazu Castellano Candalija, Germán Daroca Bengoa, Alejandro de Gea Grela, Alicia de Lorenzo Hernández, Alejandro Díez Vidal, Carmen Fernández Capitán, Maria Francisca García Iglesias, Borja González Muñoz, Carmen Rosario Herrero Gil, Juan María Herrero Martínez, Víctor Hontañón, Maria Jesús Jaras Hernández, Carlos Lahoz, Cristina Marcelo Calvo, Juan Carlos Martín Gutiérrez, Monica Martinez Prieto, Elena Martínez Robles, Araceli Menéndez Saldaña, Alberto Moreno Fernández, Jose Maria Mostaza Prieto, Ana Noblejas Mozo, Carlos Manuel Oñoro López, Esmeralda Palmier Peláez, Marina Palomar Pampyn, Maria Angustias Quesada Simón, Juan Carlos Ramos Ramos, Luis Ramos Ruperto, Aquilino Sánchez Purificación, Teresa Sancho Bueso, Raquel Sorriguieta Torre, Clara Itziar Soto Abanedes, Yeray Untoria Tabares, Marta Varas Mayoral, Julia Vásquez Manau.

C. H. U. de Albacete. Albacete

Jose Luis Beato Pérez, Maria Lourdes Sáez Méndez.

Complejo Asistencial de Segovia. Segovia

Eva María Ferreira Pasos, Daniel Monge Monge, Alba Varela García.

H. U. Puerta de Hierro. Majadahonda

María Álvarez Bello, Ane Andrés Eisenhofer, Ana Arias Milla, Isolina Baños Pérez, Javier Bilbao Garay, Silvia Blanco Alonso, Jorge Calderón Parra, Alejandro Callejas Díaz, José María Camino Salvador, Mª Cruz Carreño Hernández, Valentín Cuervas-Mons Martínez, Sara de la Fuente Moral, Miguel del Pino Jimenez, Alberto Díaz de Santiago, Itziar Diego Yagüe, Ignacio Donate Velasco, Ana María Duca, Pedro Durán del Campo, Gabriela Escudero López, Esther Expósito Palomo, Ana Fernández Cruz, Esther Fiz Benito, Andrea Fraile López, Amy Galán Gómez, Sonia García Prieto, Claudia García Rodríguez-Maimón, Miguel Ángel García Viejo, Javier Gómez Irusta, Edith Vanessa Gutiérrez Abreu, Isabel Gutiérrez Martín, Ángela Gutiérrez Rojas, Andrea Gutiérrez Villanueva, Jesús Herráiz Jiménez, Pedro Laguna del Estal, Mª Carmen Máinez Sáiz, Cristina Martín Martín, María Martínez Urbistondo, Fernando Martínez Vera, Susana Mellor Pita, Patricia Mills Sánchez, Esther Montero Hernández, Alberto Mora Vargas, Cristina Moreno López, Alfonso Ángel-Moreno Maroto, Victor Moreno-Torres Concha, Ignacio Morrás De La Torre, Elena Múñez Rubio, Ana Muñoz Gómez, Rosa Muñoz de Benito, Alejandro Muñoz Serrano, Jose María Palau Fayós, Ilduara Pintos Pascual, Antonio Ramos Martínez, Isabel Redondo Cánovas del Castillo, Alberto Roldán Montaud, Lucía Romero Imaz, Yolanda Romero Pizarro, Mónica Sánchez Santiuste, David Sánchez Órtiz, Enrique Sánchez Chica, Patricia Serrano de la Fuente, Pablo Tutor de Ureta, Ángela Valencia Alijo, Mercedes Valentín-Pastrana Aguilar, Juan Antonio Vargas Núñez, Jose Manuel Vázquez Comendador, Gema Vázquez Contreras, Carmen Vizoso Gálvez.

H. Miguel Servet. Zaragoza

Gonzalo Acebes Repiso, Uxua Asín Samper, María Aranzazu Caudevilla Martínez, José Miguel García Bruñén, Rosa García Fenoll, Jesús Javier González Igual, Laura Letona Giménez, Mónica Llorente Barrio, Luis Sáez Comet.

H. U. La Princesa. Madrid

María Aguilera García, Ester Alonso Monge, Jesús Álvarez Rodríguez, Claudia Alvarez Varela, Miquel Berniz Gòdia, Marta Briega Molina, Marta Bustamante Vega, Jose Curbelo, Alicia de las Heras Moreno, Ignacio Descalzo Godoy, Alexia Constanza Espiño Alvarez, Ignacio Fernández Martín-Caro, Alejandra Franquet López-Mosteiro, Gonzalo Galvez Marquez, María J. García Blanco, Yaiza García del Álamo Hernández, Clara García-Rayo Encina, Noemí Gilabert González, Carolina Guillamo Rodríguez, Nicolás Labrador San Martín, Manuel Molina Báez, Carmen Muñoz Delgado, Pedro Parra Caballero, Javier Pérez Serrano, Laura Rabes Rodríguez, Pablo Rodríguez Cortés, Carlos Rodriguez Franco, Emilia Roy-Vallejo, Monica Rueda Vega, Aresio Sancha Lloret, Beatriz Sánchez Moreno, Marta Sanz Alba, Jorge Serrano Ballester, Alba Somovilla, Carmen Suarez Fernández, Macarena Vargas Tirado, Almudena Villa Marti.

H. U. de A Coruña. A Coruña

Alicia Alonso Álvarez, Olaya Alonso Juarros, Ariadna Arévalo López, Carmen Casariego Castiñeira, Ana Cerezales Calviño, Marta Contreras Sánchez, Ramón Fernández Varela, Santiago J. Freire Castro, Ana Padín Trigo, Rafael Prieto Jarel, Fátima Raad Varea, Laura Ramos Alonso, Francisco Javier Sanmartín Pensado, David Vieito Porto.

H. Clinico San Carlos. Madrid

Inés Armenteros Yeguas, Javier Azaña Gómez, Julia Barrado Cuchillo, Irene Burruezo López, Noemí Cabello Clotet, Alberto E. Calvo Elías, Elpidio Calvo Manuel, Carmen María Cano de Luque, Cynthia Chocron Benbunan, Laura Dans Vilan, Ester Emilia Dubon Peralta, Vicente Estrada Pérez, Santiago Fernandez-Castelao, Marcos Oliver Fragiel Saavedra, José Luis García Klepzig, Maria del Rosario Iguarán Bermúdez, Esther Jaén Ferrer, Rubén Ángel Martín Sánchez, Manuel Méndez Bailón, Maria José Nuñez Orantos, Carolina Olmos Mata, Eva Orviz García, David Oteo Mata, Cristina Outon González, Juncal Perez-Somarriba, Pablo Pérez Mateos, Maria Esther Ramos Muñoz, Xabier Rivas Regaira, Iñigo Sagastagoitia Fornie, Alejandro Salinas Botrán, Miguel Suárez Robles, Maddalena Elena Urbano, Miguel Villar Martínez.

H. Infanta Sofía. S. S. de los Reyes

Rafael del Castillo Cantero, Rebeca Fuerte Martínez, Arturo Muñoz Blanco, José Francisco Pascual Pareja, Isabel Perales Fraile, Isabel Rábago Lorite, Llanos Soler Rangel, Inés Suárez García, Jose Luis Valle López.

Hospital Universitario Dr. Peset. Valencia

Juan Alberto Aguilera Ayllón, Arturo Artero, María del Mar Carmona Martín, María José Fabiá Valls, Maria de Mar Fernández Garcés, Ana Belén Gómez Belda, Ian López Cruz, Manuel Madrazo López, Elisabet Mateo Sanchis, Jaume Micó Gandia, Laura Piles Roger, Adela Maria Pina Belmonte, Alba Viana García.

Hospital Clínico de Santiago. Santiago de Compostela

Maria del Carmen Beceiro Abad, Maria Aurora Freire Romero, Sonia Molinos Castro, Emilio Manuel Paez Guillan, María Pazo Nuñez, Paula Maria Pesqueira Fontan.

H. Nuestra Señora del Prado. Talavera de la Reina

Sonia Casallo Blanco, Jeffrey Oskar Magallanes Gamboa.

H. U. Ramón y Cajal. Madrid

Luis Fernando Abrego Vaca, Ana Andréu Arnanz, Octavio Arce García, Marta Bajo González, Pablo Borque Sanz, Alberto Cozar Llisto, Sonia de Pedro Baena, Beatriz Del Hoyo Cuenda, María Alejandra Gamboa Osorio, Isabel García Sánchez, Andrés González García, Oscar Alberto López Cisneros, Miguel Martínez Lacalzada, Borja Merino Ortiz, Jimena Rey-García, Elisa Riera González, Cristina Sánchez Díaz, Grisell Starita Fajardo, Cecilia Suárez Carantoña, Adrian Viteri Noel, Svetlana Zhilina Zhilina.

Hospital Royo Villanova. Zaragoza

Nicolás Alcalá Rivera, Anxela Crestelo Vieitez, Esther del Corral, Jesús Díez Manglano, Isabel Fiteni Mera, Maria del Mar Garcia Andreu, Martin Gerico Aseguinolaza, Claudia Josa Laorden, Raul Martinez Murgui, Marta Teresa Matía Sanz.

H. U. Infanta Cristina. Parla

Juan Miguel Antón Santos, Ana Belén Barbero Barrera, Coralia Bueno Muiño, Ruth Calderón Hernaiz, Irene Casado Lopez, José Manuel Casas Rojo, Andrés Cortés Troncoso, Mayte de Guzmán García-Monge, Francesco Deodati, Gonzalo García Casasola Sánchez, Elena Garcia Guijarro, Davide Luordo, María Mateos González, Jose A Melero Bermejo, Lorea Roteta García, Elena Sierra Gonzalo, Javier Villanueva Martínez.

H. de Cabueñes. Gijón

Ana María Álvarez Suárez, Carlos Delgado Vergés, Rosa Fernandez-Madera Martínez, Eva Fonseca Aizpuru, Alejandro Gómez Carrasco, Cristina Helguera Amezua, Juan Francisco López Caleya, María del Mar Martínez López, Aleida Martínez Zapico, Carmen Olabuenaga Iscar, María Luisa Taboada Martínez, Lara María Tamargo Chamorro.

Hospital de Urduliz Alfredo Espinosa. Urdúliz

María Aparicio López, Asier Aranguren Arostegui, Paula Arriola Martínez, Gorka Arroita Gonzalez, Mª Soledad Azcona Losada, Miriam García Gómez, Eduardo Garcia Lopez, Amalur Iza Jiménez, Alazne Lartategi Iraurgi, Esther Martinez Becerro, Itziar Oriñuela González, Isabel María Portales Fernández, Pablo Ramirez Sánchez, Beatriz Ruiz Estévez, Cristian Vidal Núñez.

Hospital Regional Universitario de Málaga. Málaga

Mª Mar Ayala Gutiérrez, Rosa Bernal López, José Bueno Fonseca, Verónica Andrea Buonaiuto, Luis Francisco Caballero Martínez, Lidia Cobos Palacios, Clara Costo Muriel, Francis de Windt, Ana Teresa Fernandez-Truchaud Christophel, Paula García Ocaña, Ricardo Gómez Huelgas, Javier Gorospe García, Maria Dolores López Carmona, Pablo López Quirantes, Almudena López Sampalo, Elizabeth Lorenzo Hernández, Juan José Mancebo Sevilla, Jesica Martin Carmona, Luis Miguel Pérez-Belmonte, Araceli Pineda Cantero, Michele Ricci, Jaime Sanz Cánovas

H. Santa Marina. Bilbao

Maria Areses Manrique, Ainara Coduras Erdozain, Ane Elbire Labirua-Iturburu Ruiz.

H. Moisès Broggi. Sant Joan Despí

Judit Aranda Lobo, Jose Loureiro Amigo, Isabel Oriol Bermúdez, Melani Pestaña Fernández, Nicolas Rhyman, Nuria Vázquez Piqueras.

Hospital HLA Moncloa. Madrid

Guillermo Estrada, Teresa Garcia Delange, Isabel Jimenez Martinez, Carmen Martinez Cilleros, Nuria Parra Arribas.

H. del Henares. Coslada

Jesús Ballano Rodríguez-Solís, Luis Cabeza Osorio, María del Pilar Fidalgo Montero, Mª Isabel Fuentes Soriano, Erika Esperanza Lozano Rincon, Ana Martín Hermida, Jesus Martinez Carrilero, Jose Angel Pestaña Santiago, Manuel Sánchez Robledo, Patricia Sanz Rojas, Nahum Jacobo Torres Yebes, Vanessa Vento.

H. U. Torrevieja. Torrevieja

Julio César Blázquez Encinar, Joaquín Fernández-Cuervo.

H. U. La Fe. Valencia

Dafne Cabañero, María Calabuig Ballester, Pascual Císcar Fernández, Ricardo Gil Sánchez, Marta Jiménez Escrig, Cristina Marín Amela, Laura Parra Gómez, Carlos Puig Navarro, José Antonio Todolí Parra.

H. San Pedro. Logroño

Diana Alegre González, Irene Ariño Pérez de Zabalza, Sergio Arnedo Hernández, Jorge Collado Sáenz, Beatriz Dendariena, Marta Gómez del Mazo, Iratxe Martínez de Narvajas Urra, Sara Martínez Hernández, Estela Menendez Fernández, Jose Luís Peña Somovilla, Elisa Rabadán Pejenaute.

Hospital Universitario Ntra Sra Candelaria. Santa Cruz de Tenerife

Lucy Abella, Andrea Afonso Díaz, Selena Gala Aguilera Garcia, Marta Bethencourt Feria, Eduardo Mauricio Calderón Ledezma, Sara Castaño Perez, Guillermo Castro Gainett, José Manuel del Arco Delgado, Joaquín Delgado Casamayor, Diego Garcia Silvera, Alba Gómez Hidalgo, Marcelino Hayek Peraza, Carolina Hernández Carballo, Rubén Hernández Luis, Francisco Javier Herrera Herrera, Maria del Mar Lopez Gamez, Julia Marfil Daza, María José Monedero Prieto, María Blanca Monereo Muñoz, María de la Luz Padilla Salazar, Daniel Rodríguez Díaz, Alicia Tejera, Laura Torres Hernández.

H. U. San Juan de Alicante. San Juan de Alicante

David Balaz, David Bonet Tur, Carles García Cervera, David Francisco García Núñez, Vicente Giner Galvañ, Angie Gómez Uranga, Javier Guzmán Martínez, Isidro Hernández Isasi, Lourdes Lajara Villar, Juan Manuel Núñez Cruz, Sergio Palacios Fernández, Juan Jirge Peris García, Andrea Riaño Pérez, José Miguel Seguí Ripoll, Philip Wikman-Jorgensen.

H. U. San Agustin. Avilés

Andrea Álvarez García, Víctor Arenas García, Alba Barragán Mateos, Demelsa Blanco Suárez, María Caño Rubia, Jaime Casal Álvarez, David Castrodá Copa, José Ferreiro Celeiro, Natalia García Arenas, Raquel García Noriega, Joaquin Llorente García, Irene Maderuelo Riesco, Paula Martinez Garcia, Maria Jose Menendez Calderon, Diego Eduardo Olivo Aguilar, Marta Nataya Solís Marquínez, Luis Trapiella Martínez, Andrés Astur Treceño García, Juan Valdés Bécares.

H. de Mataró. Mataró

Raquel Aranega González, Ramon Boixeda, Carlos Lopera Mármol, Marta Parra Navarro, Ainhoa Rex Guzmán, Aleix Serrallonga Fustier.

H. U. Son Llàtzer. Palma de Mallorca

Andrés de la Peña Fernández, Almudena Hernández Milián.

H. Virgen de la Salud. Toledo

Ana Maria Alguacil Muñoz, Marta Blanco Fernández, Veronica Cano, Ricardo Crespo Moreno, Fernando Cuadra Garcia-Tenorio, Blanca Díaz-Tendero Nájera, Raquel Estévez González, María Paz García Butenegro, Alberto Gato Díez, Verónica Gómez Caverzaschi, Piedad María Gómez Pedraza, Julio González Moraleja, Raúl Hidalgo Carvajal, Patricia Jiménez Arandq, Raquel Labra González, Áxel Legua Caparachini, Pilar Lopez Castañeyra, Agustín Lozano Ancin, Jose Domingo Martin Garcia, Cristina Morata Romero, María Jesús Moya Saiz, Helena Moza Moríñigo, Gemma Muñiz Nicolás, Enriqueta Muñoz Platon, Filomena Oliveri, Elena Ortiz Ortiz, Raúl Perea Rafael, Pilar Redondo Galán, María Antonia Sepulveda Berrocal, Vicente Serrano Romero de Ávila, Pilar Toledano Sierra, Yamilex Urbano Aranda, Jesús Vázquez Clemente, Carmen Yera Bergua.

H. Juan Ramón Jiménez. Huelva

Francisco Javier Bejarano Luque, Francisco Javier Carrasco-Sánchez, Mercedes de Sousa Baena, Jaime Díaz Leal, Aurora Espinar Rubio, Maria Franco Huertas, Juan Antonio García Bravo, Andrés Gonzalez Macías, Encarnación Gutiérrez Jiménez, Alicia Hidalgo Jiménez, Constantino Lozano Quintero, Carmen Mancilla Reguera, Francisco Javier Martínez Marcos, Francisco Muñoz Beamud, Maria Perez Aguilera, Alícia Perez Jiménez, Virginia Rodríguez Castaño, Alvaro Sánchez de Alcazar del Río, Leire Toscano Ruiz.

H. U. Reina Sofía. Córdoba

Antonio Pablo Arenas de Larriva, Pilar Calero Espinal, Javier Delgado Lista, María Jesús Gómez Vázquez, Jose Jiménez Torres, Laura Martín Piedra, Javier Pascual Vinagre, María Elena Revelles Vílchez, Juan Luis Romero Cabrera, José David Torres Peña.

Hospital Infanta Margarita. Cabra

María Esther Guisado Espartero, Lorena Montero Rivas, Maria de la Sierra Navas Alcántara, Raimundo Tirado-Miranda.

H. U. Virgen de las Nieves. Granada

Pablo Conde Baena, Joaquin Escobar Sevilla, Laura Gallo Padilla, Patricia Gómez Ronquillo, Pablo González Bustos, María Navío Botías, Jessica Ramírez Taboada, Mar Rivero Rodrígez.

Hospital Costa del Sol. Marbella

Victoria Augustín Bandera, María Dolores Martín Escalante.

H. San Juan de la Cruz. Úbeda

Marcos Guzmán Garcia, Francisco Javier Vicente Hernández.

Complejo Asistencial Universitario de León. León

Rosario Maria García Die, Manuel Martin Regidor, Angel Luis Martínez Gonzalez, Alberto Muela Molinero, Raquel Rodríguez Díez, Beatriz Vicente Montes.

Hospital Clinic Barcelona. Barcelona

Júlia Calvo Jiménez, Aina Capdevila Reniu, Irene Carbonell De Boulle, Emmanuel Coloma Bazán, Joaquim Fernández Solà, Cristina Gabara Xancó, Joan Ribot Grabalosa, Olga Rodríguez Núñez.

C. H. U. de Ferrol. Ferrol

Hortensia Alvarez Diaz, Tamara Dalama Lopez, Estefania Martul Pego, Carmen Mella Pérez, Ana Pazos Ferro, Sabela Sánchez Trigo, Dolores Suarez Sambade, Maria Trigas Ferrin, Maria del Carmen Vázquez Friol, Laura Vilariño Maneiro.

Hospital Marina Baixa. Villajoyosa

Javier Ena, Jose Enrique Gómez Segado.

Hospital del Tajo. Aranjuez

Ruth Gonzalez Ferrer, Raquel Monsalvo Arroyo.

Hospital Insular de Gran Canaria. Las Palmas G. C.

Marina Aroza Espinar, Jorge Orihuela Martín, Carlos Jorge Ripper, Selena Santana Jiménez.

H. U. Marqués de Valdecilla. Santander

Marta Fernández-Ayala Novo, José Javier Napal Lecumberri, Nuria Puente Ruiz, Jose Riancho, Isabel Sampedro Garcia.

Hospital Torrecárdenas. Almería

Luis Felipe Díez García, Iris El Attar Acedo, Bárbara Hernandez Sierra, Carmen Mar Sánchez Cano.

H. U. Severo Ochoa. Leganés

Yolanda Casillas Viera, Lucía Cayuela Rodríguez, Carmen de Juan Alvarez, Gema Flox Benitez, Laura García Escudero, Juan Martin Torres, Patricia Moreira Escriche, Susana Plaza Canteli, M Carmen Romero Pérez.

Hospital Valle del Nalón. Riaño (Langreo)

Sara Fuente Cosío, César Manuel Gallo Álvaro, Julia Lobo García, Antía Pérez Piñeiro.

H. U. del Vinalopó. Elche

Francisco Amorós Martínez, Erika Ascuña Vásquez, Jose Carlos Escribano Stablé, Adriana Hernández Belmonte, Ana Maestre Peiró, Raquel Martínez Goñi, M. Carmen Pacheco Castellanos, Bernardino Soldan Belda, David Vicente Navarro.

Hospital Alto Guadalquivir. Andújar

Begoña Cortés Rodríguez.

H. Francesc de Borja. Gandia

Alba Camarena Molina, Simona Cioaia, Anna Ferrer Santolalia, José María Frutos Pérez, Eva Gil Tomás, Leyre Jorquer Vidal, Marina Llopis Sanchis, Mari Ángeles Martínez Pascual, Alvaro Navarro Batet, Mari Amparo Perea Ribis, Ricardo Peris Sanchez, José Manuel Querol Ribelles, Silvia Rodriguez Mercadal, Ana Ventura Esteve.

H. G. U. de Castellón. Castellón de la Plana

Jorge Andrés Soler, Marián Bennasar Remolar, Alejandro Cardenal Álvarez, Daniela Díaz Carlotti, María José Esteve Gimeno, Sergio Fabra Juana, Paula García López, María Teresa Guinot Soler, Daniela Palomo de la Sota, Guillem Pascual Castellanos, Ignacio Pérez Catalán, Celia Roig Martí, Paula Rubert Monzó, Javier Ruiz Padilla, Nuria Tornador Gaya, Jorge Usó Blasco.

H. Santa Bárbara. Soria

Marta Leon Tellez.

C. A. U. de Salamanca. Salamanca

Gloria María Alonso Claudio, Víctor Barreales Rodríguez, Cristina Carbonell Muñoz, Adela Carpio Pérez, María Victoria Coral Orbes, Daniel Encinas Sánchez, Sandra Inés Revuelta, Miguel Marcos Martín, José Ignacio Martín González, José Ángel Martín Oterino, Leticia Moralejo Alonso, Sonia Peña Balbuena, María Luisa Pérez García, Ana Ramon Prados, Beatriz Rodríguez-Alonso, Ángela Romero Alegría, Maria Sanchez Ledesma, Rosa Juana Tejera Pérez.

H. U. de Canarias. Santa Cruz de Tenerife

Julio Cesar Alvisa Negrin, José Fernando Armas González, Lourdes González Navarrete, Iballa Jiménez, María Candelaria Martín González, Miguel Nicolas Navarrete Lorite, Paula Ortega Toledo, Onán Pérez Hernández, Alina Pérez Ramírez.

C. H. U. de Badajoz. Badajoz

Rafael Aragon Lara, Inmaculada Cimadevilla Fernandez, Juan Carlos Cira García, Gema Maria García García, Julia Gonzalez Granados, Beatriz Guerrero Sánchez, Francisco Javier Monreal Periáñez, Maria Josefa Pascual Perez.

H. U. del Sureste. Arganda del Rey

Jon Cabrejas Ugartondo, Ana Belén Mancebo Plaza, Arturo Noguerado Asensio, Bethania Pérez Alves, Natalia Vicente López.

H. U. Quironsalud Madrid. Pozuelo de Alarcón (Madrid)

Pablo Guisado Vasco, Ana Roda Santacruz, Ana Valverde Muñoz.

H. de Poniente. Almería

Juan Antonio Montes Romero, Encarna Sánchez Martín, Jose Luis Serrano Carrillo de Albornoz, Manuel Jesus Soriano Pérez.

H. U. Lucus Augusti. Lugo

Raquel Gómez Méndez, Ana Rodríguez Álvarez.

H. San Pedro de Alcántara. Cáceres

Angela Agea Garcia, Javier Galán González, Luis Gámez Salazar, Eva Garcia Sardon, Antonio González Nieto, Itziar Montero Días, Selene Núñez Gaspar, Alvaro Santaella Gomez.

H. de Pozoblanco. Pozoblanco

José Nicolás Alcalá Pedrajas, Antonia Márquez García, Inés Vargas.

H. Virgen de los Lirios. Alcoy (Alicante)

Mª José Esteban Giner.

Hospital Doctor José Molina Orosa. Arrecife (Lanzarote)

Virginia Herrero García, Berta Román Bernal.

H. Nuestra Señora de Sonsoles. Ávila

Alaaeldeen Abdelhady Kishta.

H. G. U. de Elda. Elda

Carmen Cortés Saavedra, Jennifer Fernández Gómez, Borja González López, María Soledad Hernández Garrido, Ana Isabel López Amorós, Maria de los Reyes Pascual Pérez, Andrea Torregrosa García.

H. U. Puerta del Mar. Cádiz

José Antonio Girón González, Susana Fabiola Pascual Perez, Cristina Rodríguez Fernández-Viagas, Maria José Soto Cardenas.

H. Parc Tauli. Sabadell

Francisco Epelde, Isabel Torrente

Hospìtal de Montilla. Montilla

Ana Cristina Delgado Zamorano, Beatriz Gómez Marín, Adrián Montaño Martínez, Jose Luis Zambrana García.

H. Infanta Elena. Huelva

María Gloria Rojano Rivero.

H. de la Axarquía. Vélez- Málaga

Antonio Lopez Ruiz.

H. Virgen del Mar. Madrid

Thamar Capel Astrua, Paola Tatiana Garcia Giraldo, Maria Jesus Gonzalez Juarez, Victoria Marquez Fernandez, Ada Viviana Romero Echevarry.

Hospital do Salnes. Vilagarcía de Arousa

Vanesa Alende Castro, Ana María Baz Lomba, Ruth Brea Aparicio, Marta Fernandez Morales, Jesus Manuel Fernandez Villar, Maria Teresa Lopez Monteagudo, Cristina Pérez García, Lorena María Rodríguez Ferreira, Maria Begoña Valle Feijoo.

H. La Fuenfría. Cercedilla

Daniel Arregui Gallego, Jorge Blanco Briones, Gonzalo M Muzquiz Rueda, Isabel Rodríguez Fraile, Javier Rodríguez Hernández, María Ángeles Ruiz Rodríguez, Mikaela Zubillaga Gómez
